# Bone-conduction hearing implants: a potential postcode lottery

**DOI:** 10.1308/rcsann.2025.0074

**Published:** 2025-11-03

**Authors:** S Akbar, T Davies, NR Walker, G Thompson, R Fameesh, A Pahade, R Aggarwal, S Agarwal, A Muddaiah, I Anderco, E Heward

**Affiliations:** ^1^ Stepping Hill Hospital, UK; ^2^ Liverpool University Hospital Foundation Trust, UK; ^3^ Countess of Chester Hospital NHS Foundation Trust, UK; ^4^ Preston Royal Hospital, UK; ^5^ Salford Royal Hospital, UK; ^6^ Fairfield General Hospital, UK; ^7^ Blackpool Teaching Hospitals Foundation Trust, UK

**Keywords:** Otolaryngology, Hearing aids, Hearing disorders, Access to healthcare, Bone conduction hearing

## Abstract

**Background:**

Bone conduction hearing implants (BCHIs) are a valuable alternative option for patients with hearing loss when conventional hearing aids are not effective or a viable option. In the UK, specialist sites offer BCHI services. We aimed to understand whether patients face geographical barriers to accessing this healthcare service.

**Methods:**

A retrospective cohort study was performed at five hospitals in the North West of England over a one-year period (January–December 2023).

**Results:**

In total, 167 primary BCHIs were implanted (median age, 57.7 years; female, *n*=52 (31.1%)). Patients travelled a median distance of 17.3km from their home to the BCHI site. Of the patients receiving a BCHI, 108 (64.7%) lived in the locality of a BCHI site. The remaining 59 (35.3%) were referred from a non-BCHI centre. The majority of BCHIs were percutaneous (*n*=154/167, 92.2%) and were performed under local anaesthetic (*n*=127/167, 76.0%). No correlation between patient age and distance travelled was identified (*p*=0.22, *R*=−0.0951).

**Conclusions:**

The findings suggest a greater percentage of all BCHIs that are conducted are seen first at a BCHI centre initially rather than seen elsewhere. This could represent a potential geographical barrier to accessing BCHI services for patients not living in the locality of a non-BCHI providing centre. Future work is required to better understand BCHI service barriers on a national level and to identify methods to ensure equitable access for all.

## Introduction

Hearing impairment is common and leads to reduced quality of life. In selected cases, bone conduction hearing implants (BCHIs) can optimise hearing to reduce disability. BCHIs deliver sound to the cochlea via vibration of the skull, allowing the device to be placed on the cranium away from the external auditory canal.^1^ BCHIs are used to rehabilitate hearing in patients who cannot used conventional air conduction hearing aids. Criteria for use of BCHIs are outlined in the NHS Clinical Commissioning Policy.^2^

Bone conduction hearing aid devices have gained increasing popularity and have been shown to be an effective treatment for patients with hearing loss.^3^ Both transcutaneous and percutaneous BCHI devices can be implanted by the NHS. Approximately 800 BCHIs are fitted in the UK each year.^4^

NHS commissioning guidance outlines that BCHIs should be undertaken at a specialist auditory implant centre. Accessibility challenges have been reported where specialist surgical services are centralised.^5^ While data exist internationally that indicate that proximity to a specialist auditory implant centre does not impact upon timing or availability of auditory implantation, no such data exist outlining this in the UK.^6^

We aim to establish whether geographical healthcare barriers exist for patients eligible to access BCHI services in the North West of England.

## Methods

Strengthening the Reporting of Observational Studies in Epidemiology (STROBE) guidelines were utilised for this body of work.

### Study design

A retrospective cohort study was performed at five BCHI sites in the North West of England. Adult patients were included from 1 January 2023 to 31 December 2023. Data from each BCHI site were collected using Microsoft Excel on NHS secure encrypted computers. A ‘heat map’ was constructed with patient home postcodes. Distance in kilometres travelled by each patient to the hospital site in which the surgery took place was calculated as the shortest driving route on Google Maps.

All participant identifiable and/or sensitive data were fully anonymised. The Health Research Authority decision tool did not indicate ethical review was required.^7^ This quality improvement project was registered at each participating site.

### Setting

The North West of England region is the second most densely populated geographical area in the UK, with an average of 525 people per square kilometre in 2023.^8^ The patient’s local hospital (with ENT and audiology services available) was defined by using the patient’s postcode to identify which hospital catchment area it belonged to. If there was crossover of catchment area then the geographically closest hospital was used. Referral letters were also used to determine whether patients were referred from a non-BCHI site.

Five hospital sites from four hospital trusts in the North West of England region were involved in the study: Blackpool Victoria Hospital, The Countess of Chester Hospital, Fairfield General Hospital, Royal Preston Hospital and Salford Royal Hospital. In the North West of England there are five hospital trusts with BCHI services and nine trusts without BCHI services.

### Participants

Adult patients over the age of 16 years undergoing a primary BCHI insertion were included.

### Variables

Patient demographics and surgical details were collected. The patient’s postcode was used to determine the distance from BCHI site and whether the BCHI was carried out at their local hospital.

### Statistical analysis

A Pearsons correlation was used to look at the association between distance to BCHI centre against patient’s age. Alpha significance value was <0.05.

## Results

A total of 167 patients underwent a BCHI procedure from 1 January 2023 to 31 December 2023 (Blackpool Victoria Hospital *N*=19; Countess of Chester Hospital NHS Foundation Trust *N*=35; Fairfield General Hospital *N*=43; Royal Preston Hospital *N*=45; Salford Royal NHS Foundation Trust *N*=25).

**Figure 1:**
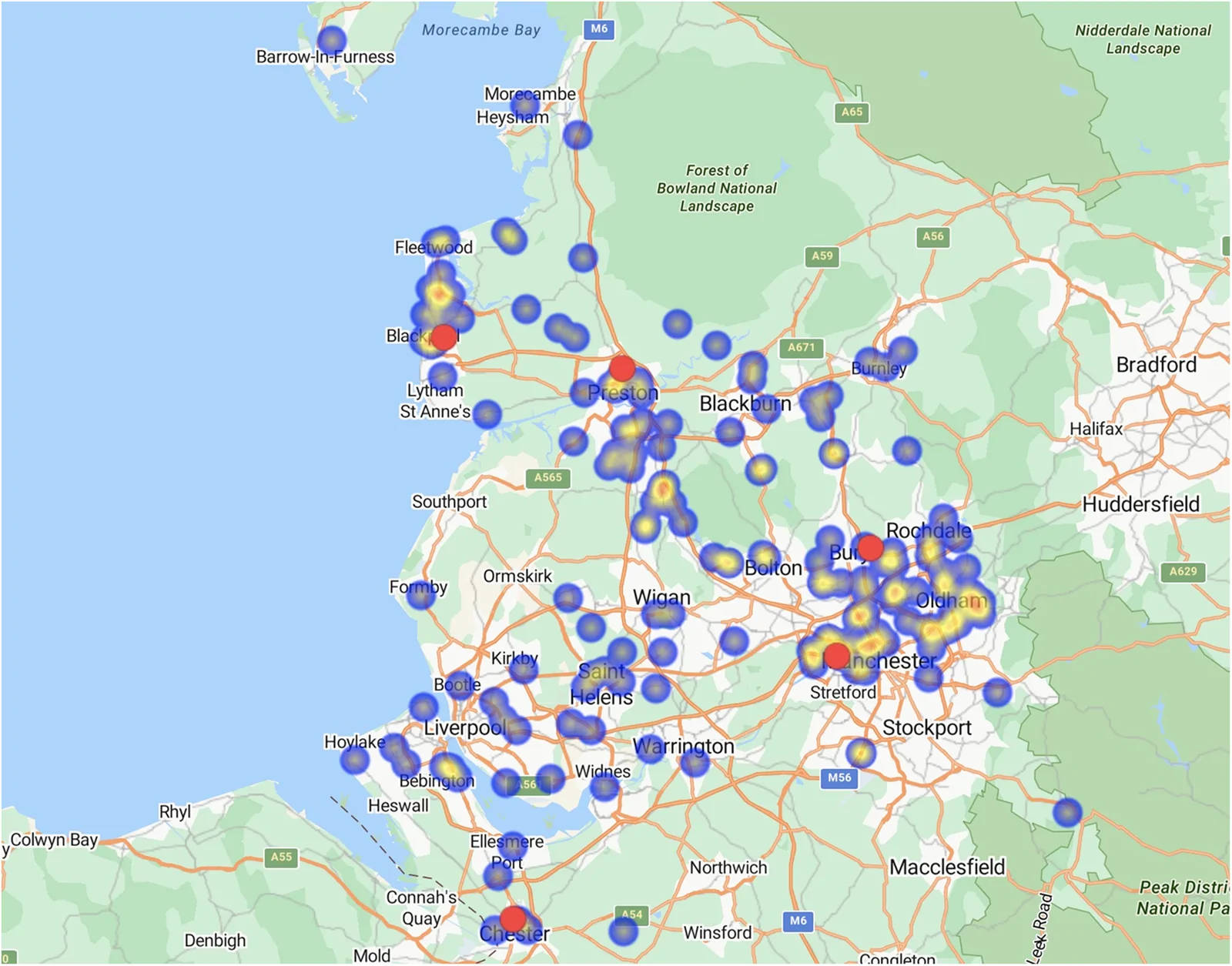
Heat map showing distribution of patients undergoing BCHI and hospital sites. The five BCHI sites included in the study are indicated by red dots on the map. High density of patients in the same geographical area is represented by orange shading. Reducing density is represented by yellow and blue shading. BCHI = Bone conduction hearing implants.

**Figure 2:**
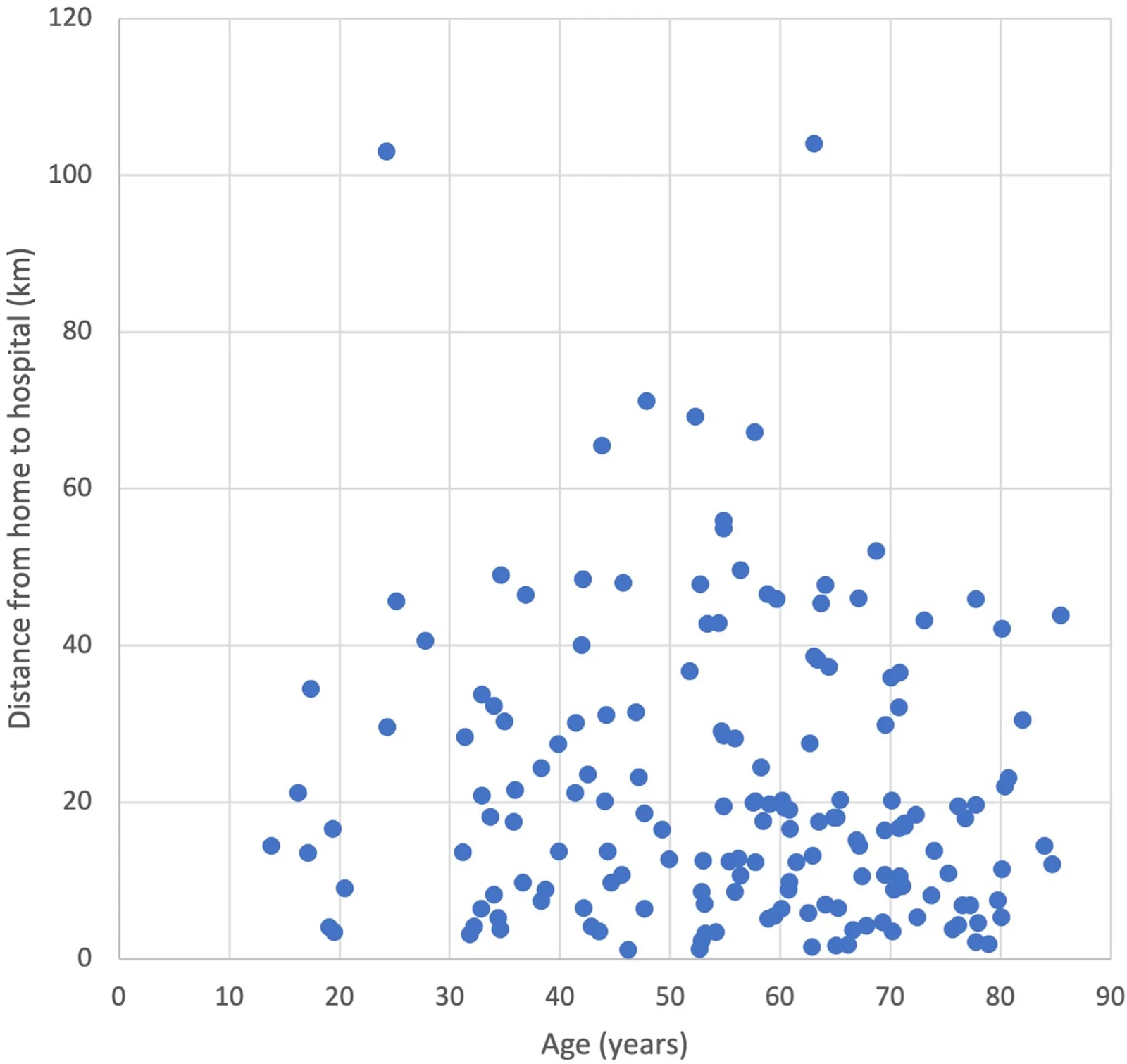
Distance travelled to BCHI site against age

Participant median age was calculated as 57.7 years and 31.1% were female. Patients travelled a median distance of 17.3km from their home to the BCHI site (Figure 1). Of those patients receiving a BCHI, 108 patients (64.7%) lived in the locality of the BCHI site. The remaining 59 (35.3%) were referred to a BCHI centre for treatment. There was no correlation between distance travelled to BCHI site and age (*p*=0.222, *R*=−0.0951) (Figure 2).

Percutaneous BCHIs were implanted most frequently (92.2%, *n*=154), with 13 transcutaneous devices used. The majority of patients (63.9%) had a right-sided operation.

Local anaesthesia was used for 76.0% of patients, with 34.0% of patients undergoing general anaesthetic. In 92.2% of cases, the BCHI insertion was the only procedure performed. Other procedures included: examination under anaesthetic of the ears (*n*=5), grommet (*n*=3), mastoid exploration (*n*=2), labyrinthectomy (*n*=1) and meatoplasty (*n*=1).

## Discussion

This is the first UK study to investigate geographical barriers to BCHI service access. Our findings suggest that patients who live in the locality of a BCHI centre have a higher likelihood of being able to access BCHI services. A similar phenomenon has been observed for specialist procedures offered by orthopaedics, plastic surgery and vascular surgery.^9–11^ Within the remit of otolaryngology, inequality in access to cochlear implants has also been described previously in the UK.^12^

The reasons for reduced access for those living outside the locality of a BCHI centre are likely multifactorial. Evidence suggests that engagement with healthcare services decreases with increasing distance.^13^ This could be related to increased financial and time burden on patients. The socioeconomic backgrounds of patients living in the North West of England are varied. Patients who have to travel to a more distant hospital site may encounter barriers such as public transport and lost working days. Efforts should be made to mitigate barriers, such as by conducting audiological assessment and ENT review on the same day and reducing unnecessary postoperative appointments. Hospital transport should be available for those needing to travel further than their local hospital for treatment. Further work is needed to establish the impact of patient socioeconomic status on BHCI uptake.

Furthermore, patients seen at a non-BCHI centre require referral to an alternate site for assessment and potential treatment, which adds additional appointments for this population. To address this, it may be possible for high-volume sites to introduce multidisciplinary or virtual clinics. It is also likely that medical professionals at non-BCHI sites are less familiar with BCHIs and their inclusion criteria, preventing identification of potential candidates. The British Cochlear Implant Group has developed a champions scheme where a named audiologist in each UK department is responsible for ensuring patients eligible for cochlear implantation are informed about their options, referred in a timely manner, and that links are developed with the implant centre. It may well be prudent to develop such a scheme for BCHIs to increase uptake at non-BCHI centres.

There is a large variation in BCHI practice across the UK.^14^ The Greater Manchester, Cumbria and Lancashire, Cheshire and Merseyside health authorities that are included in this study are known to have a higher than national average BCHI implantation rate.^12^ It is difficult to determine whether the rate of BCHI implantation has changed compared with the data from Gaunt et al. from 2008–2010 due to only primary BCHIs being included and all but one site in the region enrolled in the study.^14^ As expected, the majority of BCHIs implanted were percutaneous devices, with only 13 transcutaneous implants fitted. Percutaneous implants are placed most frequently under local anaesthetic, which is well tolerated and avoids anaesthetic-related risks, and the average age is 57.7 years. It is interesting that 63.9% of patients had a right-sided implant, which could reflect that most people are right-hand dominant. Very few BCHIs were fitted concomitantly with other procedures, perhaps to help determine postoperative hearing thresholds and determine the suitability of BCHIs.

This study is limited to the experience and patient population in the North West of England. Distance travelled to each site is relevant to the regional geography and will not be generalisable to all UK regions. The patient populations included in this research are from both urban and rural settings. Analysis using median values has aimed to reduce the impact of outliers who may travel very short (urban) or long (rural) distances. Not all BCHI centres from the North West of England took part in this study (*N*=5/6), which prevented population-based analysis. The heatmap displayed as Figure 1 is a visual representation of the population distribution. It is assumed that patients are referred to their nearest ENT department from primary care, which is not always the case. To mitigate this bias, the authors have used a pragmatic definition to determine the patient’s local hospital and screened referral letters to identify patients referred from non-BCHI sites. An additional limitation of this study is that the use of nonsurgical bone-anchored hearing aids was not included, so their use patterns in patients eligible for BHCI was not established.

Future studies should look to incorporate geospatial modelling to understand the patterns of geographical referrals in this patient population, identification of the total number of eligible patients for BCHIs compared with the number of BCHIs performed, and to explore patient opinions in relation to BCHI access barriers.

## Conclusions

Findings suggest a greater percentage of all BCHIs that are conducted are seen first at a BCHI centre rather than seen elsewhere. Methods to overcome geographical barriers to BCHI services must be identified to enable equitable access to hearing rehabilitation. These changes could have a significant impact on hearing-related disability.
